# Subjective individuals’ perception during evacuation in road tunnels: Post-experiment survey results

**DOI:** 10.1371/journal.pone.0283461

**Published:** 2023-03-30

**Authors:** Natalia Schmidt-Polończyk

**Affiliations:** Faculty of Civil Engineering and Resource Management, AGH University of Science and Technology, Kraków, Poland; Al Mansour University College-Baghdad-Iraq, IRAQ

## Abstract

The aim of the research was to analyse the process of evacuation from the point of view of the individual’s perception, behaviour and decision making. The study used a survey method that was conducted during two real-scale evacuation experiments in real road tunnels under smoky conditions. All experiments, with fire scenarios and procedures were very similar to real accident. Respondents’ observations and important aspects affecting the evacuation process were verified, including decision-making during evacuation, loss of bearing in smoky conditions and group evacuation. The results indicate that participants in the experiments had started the evacuation due to smoke in the tunnel and fire drill. The evacuees observed decreased visibility on the escape route as well as a loss of bearing in the tunnel when smoke levels were high (extinction coefficient Cs > 0,7 m^-1)^. The participants in the experiment evacuated in a group (when the tunnel infrastructure was unknown and there was no instruction as to what to do) and in twos under the smokiest conditions (extinction coefficient Cs~1.0–1.1m^-1^). During the experiments, the large impact of herding behaviour and following the group was noticed. The results of such studies based on real-scale evacuation experiments in road tunnels are essential to improve the level of safety in the road tunnel. In the surveys, the participants pointed to important issues related to evacuation, which require particular attention during the design, implementation and acceptance of this type of construction. The results of the study provide a better understanding of the behaviour of evacuees and indicate areas where tunnel infrastructure needs to be improved.

## 1 Introduction

Road tunnels are an increasingly important element of road infrastructure, so the safety aspect in these facilities has become a frequent subject of scientific research. From a statistical point of view, the accident rate in tunnels is lower than in the case of open roads. Nevertheless, it is noteworthy that due to the geometrical characteristics of road tunnels, the effects of hazardous situations occurring in road tunnels may be on a much greater scale than those occurring in open road spaces. On the open roads the factors which increase the likelihood of serious injuries in case of an accident with a truck include limited lighting or speed above 60 km/h. These and other harmful factors, such as limited space, are characteristic of road tunnels, which increases the likelihood of severity of the harm [1]. Moreover, the rise in hydrogen fuel cell electric vehicles (FCEVs) is expected to pose a variety of hazards in the road tunnels [2].

One of the most critical situations in tunnels are fires which may occur. This is evidenced by their tragic consequences, e.g. in the Mont Blanc tunnel in 1999, where 39 people died, mainly because these people failed to evacuate, probably due to unawareness of the danger and ignorance of safety procedures. A similar scenario occurred during the 2006 Viamala tunnel fire in Switzerland, which resulted in nine deaths; the 2010 Wuxi Lihu tunnel fire, where there were 24 fatalities; the 2014 Yanhou tunnel fire in China, which resulted in 40 deaths; or, for example, the 2017 Taojiakuang tunnel fire in Weihai, China. (12 fatalities)—these are only selected examples [3]. This is when immediate and efficient evacuation is the most important issue for protecting human health and life. The outcome of this process is dependent on a range of factors, including: technical infrastructure [4–7], tunnel equipment [8–10], safety procedures [11–13], training of personnel responsible for safety in tunnels and rescue services training sessions [14] as well as the human factor. In order to conduct a safe and effective evacuation, the key issue for the evacuees is to get information about the evacuation details (such as the information about the danger and the direction of evacuation) [15].

The human perception of road tunnel infrastructure during fires is of particular interest in fire safety engineering, especially when the consequences of accidents in partially enclosed spaces, such as tunnels, can be much more severe [16]. To improve safety in road tunnels various methodologies are used. One can point to studies based on real-case analyses [17, 18], experimental studies [19–25], surveys and computer simulation studies [26, 27]. It should be stressed that the behavioral aspects of the evacuation process have become an increasingly more important subject of research over the past few years [22, 17, 28–30].

On the basis of two real scale evacuation experiments in smoky conditions, the individual perception during the evacuation process has been studied. For this purpose, the survey results methodology was used (the results were recorded directly after each experiment). The post-experiment questionnaire includes a number of questions designed to verify respondents’ observations and important aspects affecting their decision during evacuation.

The main objectives of the surveys performed during experiments can be pointed out as follows:

- decision making during the evacuation,
- influence of smoke on individuals’ behavior,
- participants’ well-being during experiments,
- participants’ observations (about: tunnel infrastructure, emergency systems, their own behaviors).

The article is organized in the following order: Section 2 presents research methodology and description of two real scale experiments; survey results are presented in Section 3. Finally, Section 4 includes discussion of results and concludes the research, while references are included Supplementary material, for example the survey is included in the S1 Appendix.

These are the first and only full-scale experiments in Poland run on such a high level of authenticity—no previous research succeeded in creating the situation of fire emergency where the circumstances would be as actual as they can be. The use of smoke, causing the loss of visibility and confusion among participants, along with the application of real emergency procedures, created a situation in which the individual perception of people facing an emergency can be studied.

## 2 Research methodology

Individual perceptions included in the post-experiment questionnaire have been analyzed in this study. The answers of the 140 participants, who took part in real scale experiments of evacuation in fire conditions in road tunnels, were taken into consideration. The participants were asked to complete a survey about their observations and impressions during the evacuation.

The survey is composed of two parts (see S1 Appendix). Part One includes census data: age, gender, education and parameters like height, weight and shoulder width. Additionally, participants were asked about previous experiences with evacuation and smoke conditions. Part Two asks questions related to: decision making during the evacuation, the wellness of evacuees, loss of bearing in smoky conditions, evacuation in groups and the influence of smoke on individuals behaviour. Participants were also encouraged to write down their observations and impressions from each experiment. The second part of survey consists of 2 multiple choice questions, 12 single choice questions (10 after experiment 1) and one open question for participants’ observation.

The surveys were completed directly after each trial of both experiments in Laliki and Gdańsk.

### 2.1 Description of real scale experiments

A set of evacuation experiments were performed in two road tunnels in Poland: in the Emilia Tunnel in Laliki and in the tunnel under the Dead Vistula River in Gdańsk. The main objectives of these experiments were to estimate human behavior and interaction during evacuation and to test how pedestrians react when exposed to reduced visibility and how the decision making process is carried out.

The participants that took part in the both evacuation experiments were recruited in a two-step process. Firstly, they were informed about the possibility of participating in the evacuation experiment, without giving any details. In Laliki, 50 students of the AGH University of Science and Technology were involved; in Gdańsk, information about the experiment in local media, radio and social media were given. In the second phase, health and other contraindications for participating in the experiment were checked. In Laliki, students had had a medical statement in order to participate in an industrial internship; in Gdańsk, individuals were interviewed about their health, mental and physical condition. Additionally, their sobriety was checked. All participants took part in both experiments voluntarily and signed the statement accordingly. Both evacuation experiments were conducted using non-toxic cold smoke to create experimental conditions which would reflect, as accurately as possible, the conditions occurring during a fire, the greatest threat associated with tunnel use. In both cases big group of people assembled in a bus evacuated in a road tunnel in smoky conditions. All scenarios and their infrastructure were very similar to real accident including: presence of smoke, activation of the emergency procedures in the tunnel such as the alarm, voice communications and emergency lights. It should be highlighted that the participants did not know the purpose or scenario and were unfamiliar with the tunnel infrastructure. Their reaction to the tunnel infrastructure in fire conditions during experiments might have been very similar to a real accident. Furthermore, before entering the tunnel, the participants did not know that the evacuation experiment would involve smoke in the tunnel.

#### 2.1.1 Tunnel Emilia in Laliki

**Fig 1:**
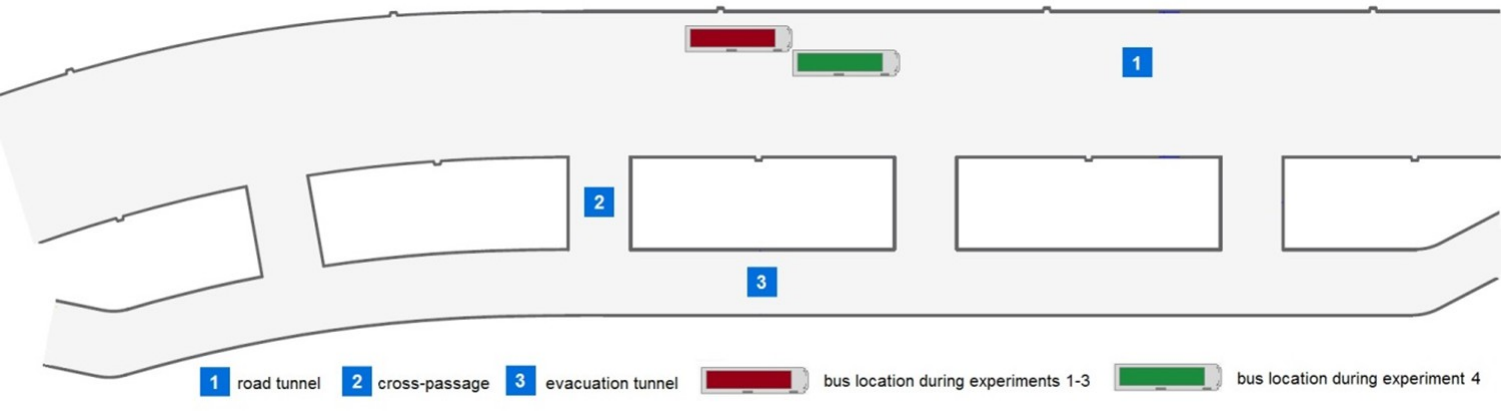
Bus location during experiments 1–4 in Emilia tunnel in Laliki.

The first experimental research was carried out in Emilia: a 678 m long road tunnel located in south-west Poland on October 19, 2016. The tunnel comprises two parallel tubes: one tube of a bidirectional road tunnel with two traffic lanes and an evacuation tube. There are four cross passages every 150 m, connecting the road tunnel with the evacuation tunnel (Fig 1).

**Fig 2:**
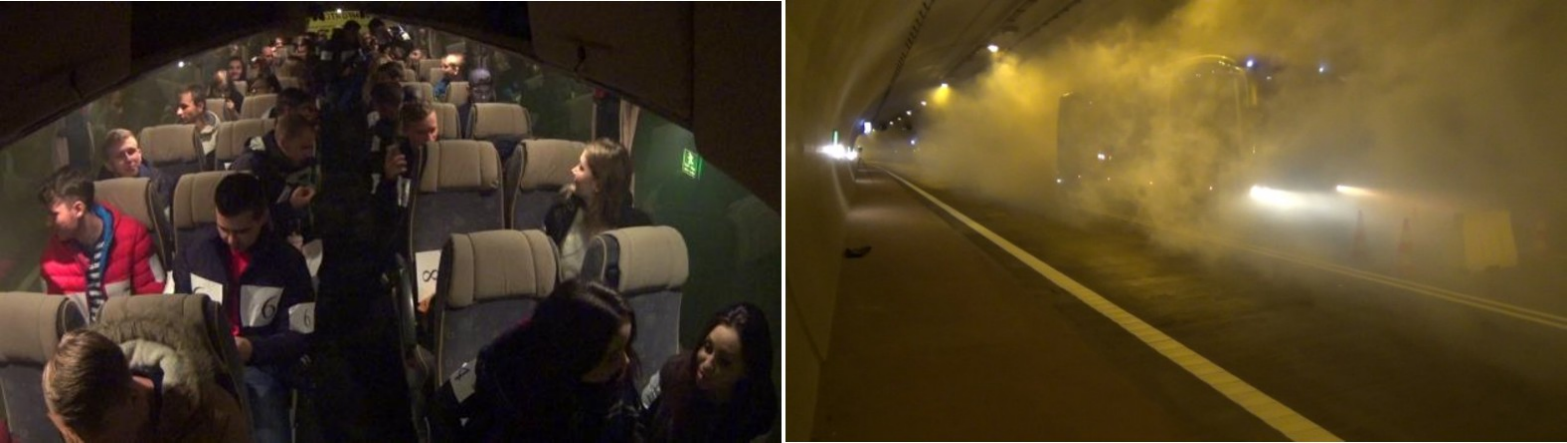
Participants of an experiment in a road tunnel in Laliki, a bus stopped in a smoky tunnel before the evacuation started.

The experiment consisted of four trials, and approximately 50 students of the AGH University of Science and Technology took part in this research. The group consisted of 34 males and 16 females, with average age 21 years (the age span in this case is 19 to 24 years). In Experiment 1 (with a low level of smokiness–the extinction coefficient was Cs≤0.2 m^−1^ (the extinction coefficient based on the Lambert–Beer equation)) [31]; the participants did not receive any instructions as to the purpose of the experiment, how to behave in case of smoke or any information about the infrastructure of the tunnel (the location of the evacuation cross—passages as well as the position of the bus in the tunnel). The bus was directed into the tunnel without stopping, in order to ensure an element of surprise for the participants. The position of the bus in this experiment was in the middle of the tunnel, exactly as in the Experiments 2 and 3. In Experiment 4, the vehicle stop location was changed; therefore, in this case, the issue analyzed was the choice of evacuation route under difficult conditions of very poor visibility (Cs ≥0.9 m^−1^)—Table 1. In each case, a vehicle carrying passengers was stopped in a tunnel and artificial smoke was generated around them (Fig 2). Then, the passengers initialized evacuation from the vehicle and the smoke filled road tunnel.

**Table 1:** List of experiments with different levels of visibility, tunnel familiarity of group of participants evacuated and expressed task.

Number	Level of smokiness	Tunnel Familiarity	Task for passengers
Experiment 1	Light smoke ~0.1–0.2m^-1^	No	No direct, expressed task
Experiment 2	Moderate smoke ~0.4–0.5m^-1^	Yes	To evacuate
Experiment 3	Heavy smoke ~0.8–0.9m^-1^	Yes	To achieve the best time
Experiment 4	Heavy smoke ~1.0–1.1m^-1^	Yes	To evacuate

#### 2.1.2 Tunnel under the Dead Vistula River in Gdańsk

**Fig 3:**
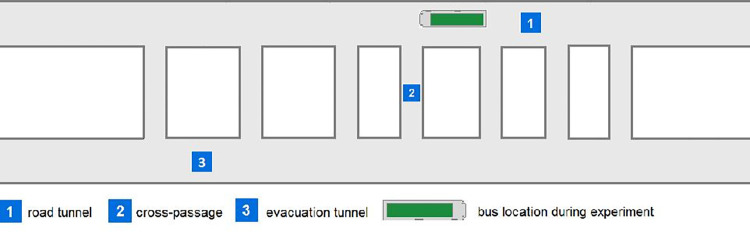
Bus location during experiments 1–2 in the tunnel under the Dead Vistula River in Gdańsk.

The other evacuation experiment, consisting of three experiments, was performed in June 2017 in the tunnel under the Dead Vistula River in Gdańsk (1378 m in length). The tunnel comprises two parallel tubes, each with two traffic lanes in one direction. There are seven evacuation exits distanced from 115 m up to 175 m, connecting two tunnels (Fig 3). In case of fire in one tunnel, the second tube is dedicated to safe evacuation.

The evacuation experiment involved 90 participants—61 males and 29 females, with an average age of 27 years (the age span in this case is 12 to 49 years). Firstly, passengers have to leave the bus and secondly, they have to evacuate from the tunnel with fire. Experiments were performed in heavy smokiness (extinction coefficient Cs>0.7m^−1^) but with non-toxic, non-irritant smoke in order to ensure safe conditions.

**Fig 4:**
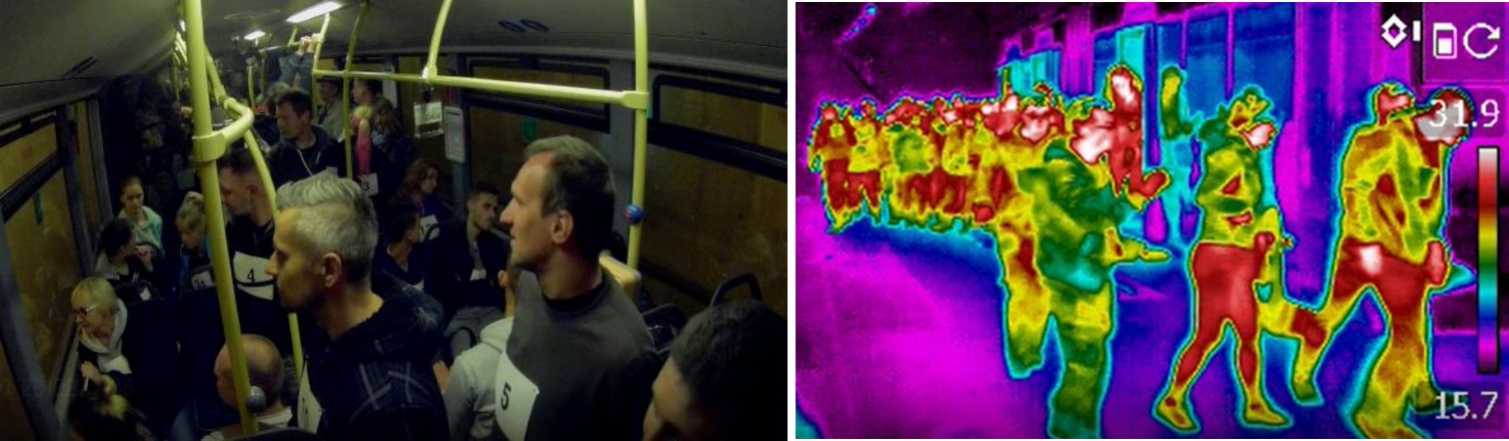
Participants in the bus and main tunnel during one of the experiments in Gdańsk.

**Fig 5:**
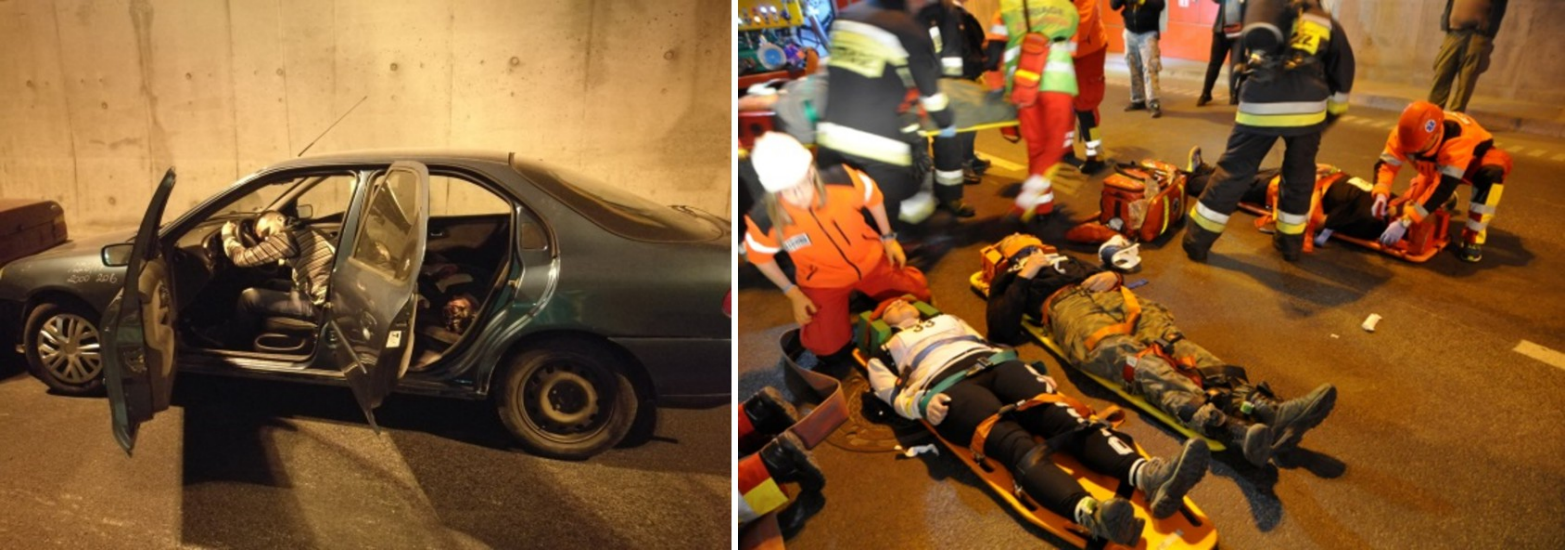
Experiment 3 in Gdańsk, dedicated to fire brigade and rescue service exercises.

During the research, three experiments were performed, but the first two became the source of data used in this study (Fig 4). The last part was dedicated to fire brigade and rescue service exercises (Fig 5). Moreover, during all trials involved in the experiment, two car wrecks simulated a collision of vehicles.

### 2.2 Ethics statement and setting

During the first study conducted with human participation in the tunnel Emilia in Laliki, the opinion of the Bioethics Committee in District Medical Chamber in Krakow, in Poland was asked regarding the ethical aspects of the research. The Committee stated that in case of such circumstances, no special consent of the Ethics Committee is required. Prior to the second experiment in Gdańsk, the Committee gave their oral approval.

Both experiments were conducted in the same circumstances:

- it was under a constant supervision of the fire brigade, the police, and other security services, as well as the organizers of the experiment and the tunnel management,
- the smoke used to imitate the real fire emergency was cold, non-irritating and non-toxic,
- all the safety procedures were maintained, and the security measures such as smoke emergency lighting were applied,
- the participation was voluntary and the participants signed a written consent.

What is more, prior to the experiment in Gdańsk, the medical services conducted a medical interview and check-up to exclude any potential contraindications, which also included the sobriety of participants. The experiment was run only after all of the health criteria were checked and verified, and any potential dangers excluded.

## 3 Individual’s perception–survey results

### 3.1 Decision making during the evacuation

The decision process to start evacuation was analyzed on the basis of the first experiment in the Laliki and Gdańsk tunnels. Both these first experiments were the only ones in which individuals were unfamiliar with the tunnels’ infrastructure and did not expect these situations. Pedestrians only knew that they would take part in an evacuation experiment in a road tunnel. They were not informed about the details of the experiment and did not expect any artificial smoke, sirens, emergency lights or other emergency tunnel infrastructure. The bus drivers were instructed to remain in their seats and not to give any prompts to participants, except the opening doors, when they heard the voice alarm. The scenario for both tunnel experiments was very similar: a bus with individuals (passengers) inside entered the road tunnel and stopped. Then, smoke began to surround the bus; moments later, a siren was heard followed by voice alarm messages in Polish and English, and emergency lights were activated simultaneously.

After the individuals evacuated, the respondents’ perception of the evacuation process, which mostly affected their decisions, were checked in the post-experiment survey.

**Fig 6:**
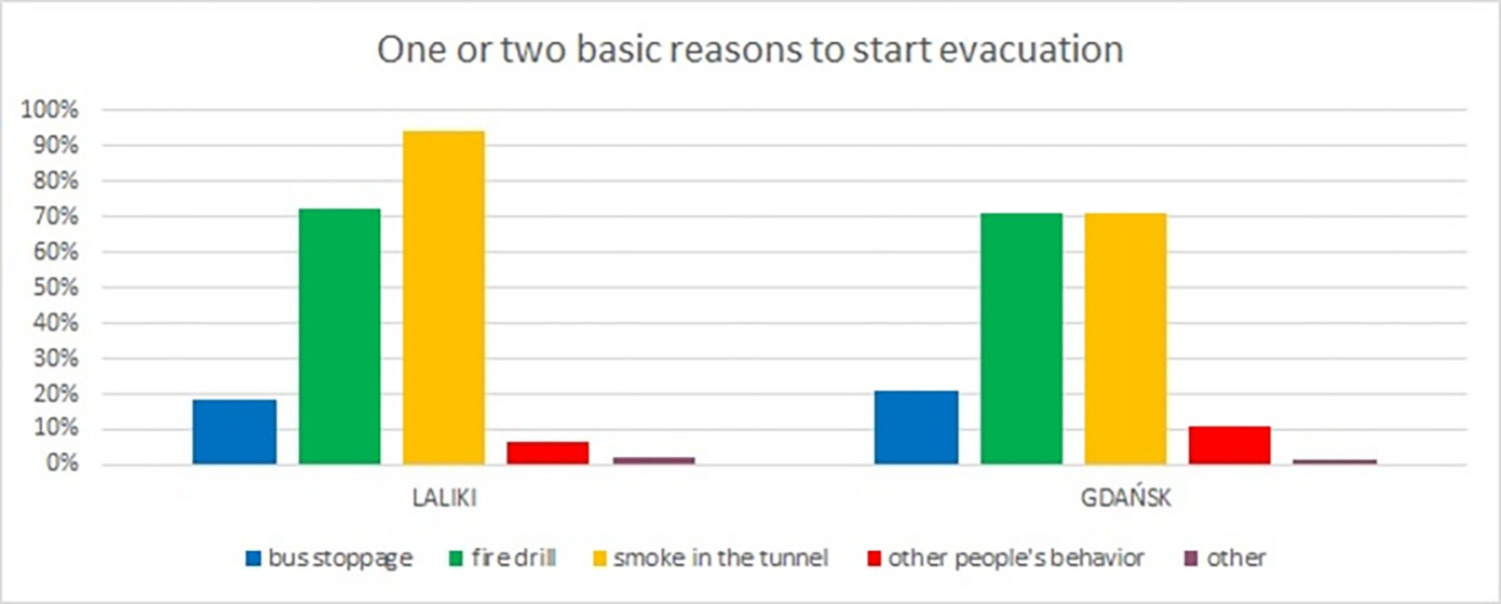
Survey results in question about reasons for decision to start evacuation (results from Experiment 1).

**Fig 7:**
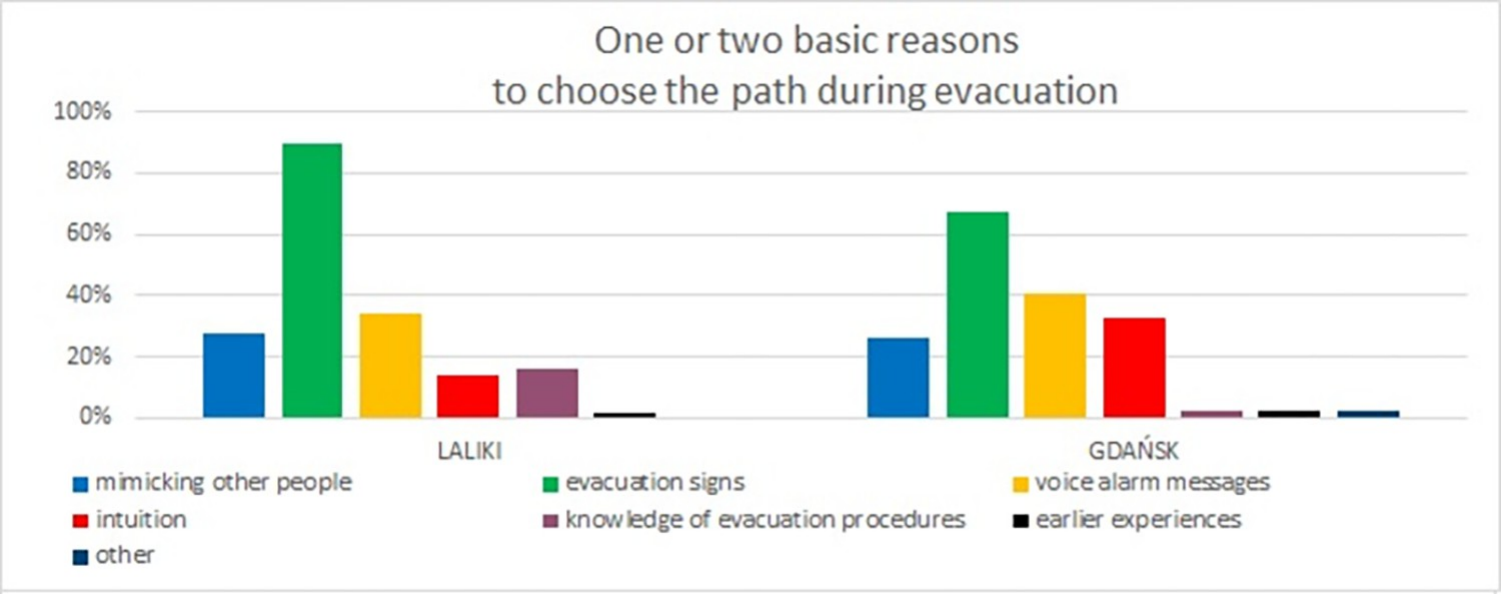
Reasons for choosing a specific evacuation path (results from Experiment 1).

In the first two questions, participants were asked about the reasons for their decision to start evacuating (Fig 6) and to choose an evacuation path (Fig 7). In the first question, participants answered that they started evacuation mostly due to smoke in the tunnel (Laliki: 94%, Gdańsk: 71%) and the fire drill (Laliki: 72%, Gdańsk: 71%), which included a fire siren and voice message. Some persons also indicated that the bus stopping was an important factor. Other reasons are omitted.

In question 2, participants in both experiments indicated that their main reason for choosing a particular evacuation path was the influence of evacuation signs (Laliki: 90%, Gdańsk: 68%). In second place were voice alarm messages (Laliki: 34%, Gdańsk: 40%). The third most common answer overall was mimicking other people (chosen by 28% of respondents from Laliki and 27% from Gdańsk). Similarly, 14% of the participants from Laliki and 33% of those from Gdańsk indicated intuition (Fig 7).

**Fig 8:**
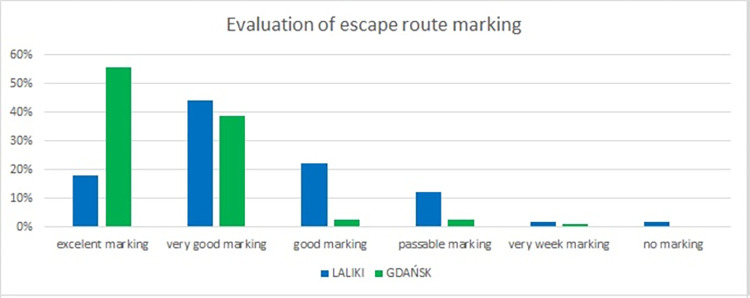
Pedestrians’ estimation of the escape route marking (results from Experiment 1).

**Fig 9:**
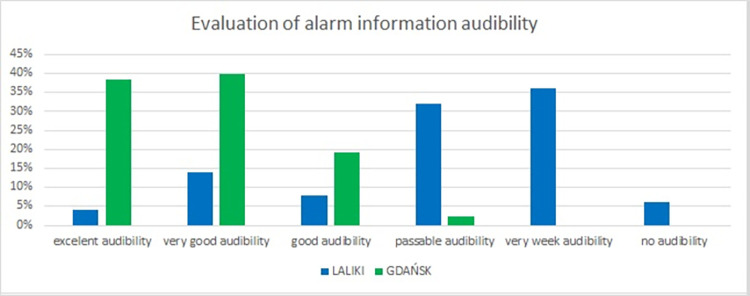
Pedestrians’ estimation of the audibility of the alarm message in the tunnel (prerecorded) (results from Experiment 1).

In questions 11 and 12, we asked participants to rate alarm information audibility and escape route marking (Figs 8 and 9). In Laliki, tunnel marking of evacuation paths was rated as very good by 44% respondents, good by 22% and excellent by 18%. The three lowest scores (passable, very weak and no marking) were rarely given (total–Laliki: 16%). In the Gdańsk tunnel, 55% of individuals rated escape route marking as excellent and 39% as very good–Fig 8. Regarding participants’ evaluation of alarm information audibility in Laliki and Gdańsk tunnels when participants heard this information for the first time: in Laliki, most answers were *very weak* (36%) and *passable audibility* (32%)–in sharp contrast to Gdańsk, where alarm information audibility was rated as *very good* (40%) or *excellent* (39%) (Fig 9).

**Fig 10:**
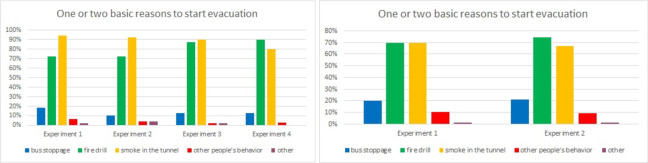
Survey results in question about decision premises to start evacuation–question 1. (Results from Laliki experiment are on the left side, from Gdańsk on right).

As mentioned earlier, in the Experiment 1 in Laliki and in Gdańsk, the participants did not receive any instructions as to the purpose of the experiment (such as suggested behavior or tunnel infrastructure). These experiments were the most significant with regard to the decision to start evacuating and choice of exit, mainly because there was an element of surprise for participants, as in a real fire. In the questionnaires, individuals said they had started to evacuate because of *smoke in the tunnel* and *the fire drill*. These answers in other trials, when individuals were familiar with the scenario and tunnel infrastructure, were also checked. In all four experiments in Laliki and also two in Gdańsk, respondents were unanimous (Fig 10).

**Fig 11:**
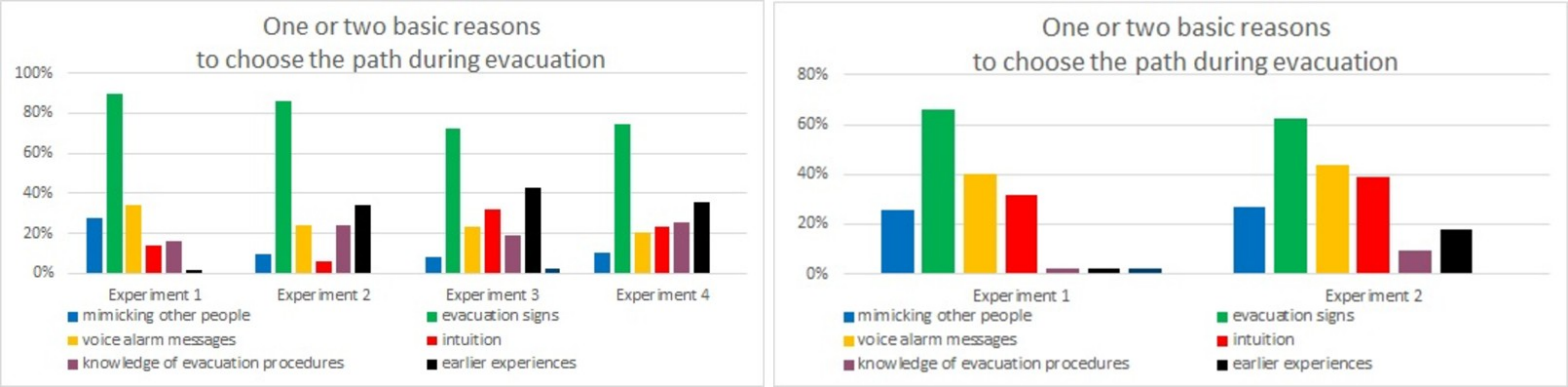
Reasons to choose a specific evacuation path–question 2. (Results from Laliki experiment are on the left; results from Gdańsk are on the right).

All survey experiment results for the main reasons for choosing a particular path during evacuation have been analyzed and compared. The majority of respondents seem to have mostly appreciated the *evacuation signs* (Laliki average score—81%, Gdańsk—64%). 37% of participants in the Laliki experiment also indicated *previous experiences* (average score in trial 2,3 and 4), 25% *voice alarm messages* (average score) and 21% *knowledge of evacuation procedures* (Fig 11). In Gdańsk, 42% of individuals pointed to the *voice alarm messages*, 35% *intuition* and 26% *mimicking other people* (Fig 11). It seems that choice of exit is important *evacuation signs* and *voice alarm messages* are helpful.

### 3.2 Influence of smoke on individuals behavior

**Fig 12:**
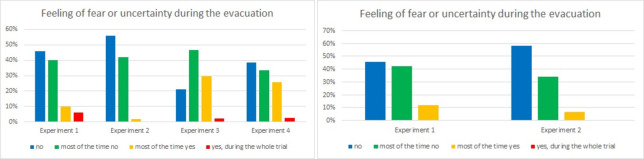
Feeling of fear or uncertainty among evacuees during consecutive experiments–question 3. (Results from Laliki experiment are on the left; results from Gdańsk are on the right).

The series of four questions is intended to investigate the influence of smoke on participants’ behavior. In question 3 evacuees, responded as to whether they felt any fear or uncertainty (Fig 12).

In Laliki, during Experiment 2, evacuees felt the safest and most confident—56% said no, and 42% said most of the time no; in this experiment, the smokiness level was moderated and the participants were aware of what would happen. Participants felt more confident during Experiment 1 when they unexpectedly found themselves in an alarm drill, than in Experiments 3 and 4 when they had to move in heavy smoke.

In Laliki, in first experiment, the answers were: (no—46%, most of time no—40%, most of time yes– 10,00%, yes, during whole trial– 6,00%); in the third and fourth experiments, the answers were 21%, 47%, 30%, 2% and 39%, 33%, 26%, 2% respectively. This may suggest that lack of visibility is a more likely reason for fear and discomfort than an unexpected situation and sudden need of evacuation. In Gdańsk, both experiments were conducted at similar levels of smokiness, which may explain why the results are similar, while in the second experiment, almost 60% of pedestrians did not feel any fear or uncertainty.

**Fig 13:**
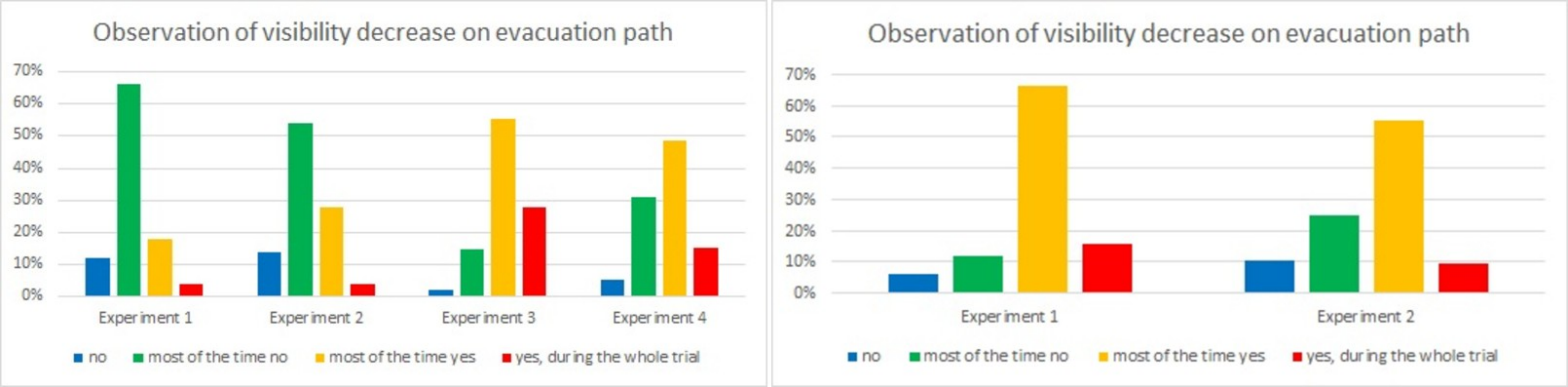
Pedestrians’ observations on visibility loss—question 4. (Results from the Laliki experiment are on the left; results from Gdańsk on right).

**Fig 14:**
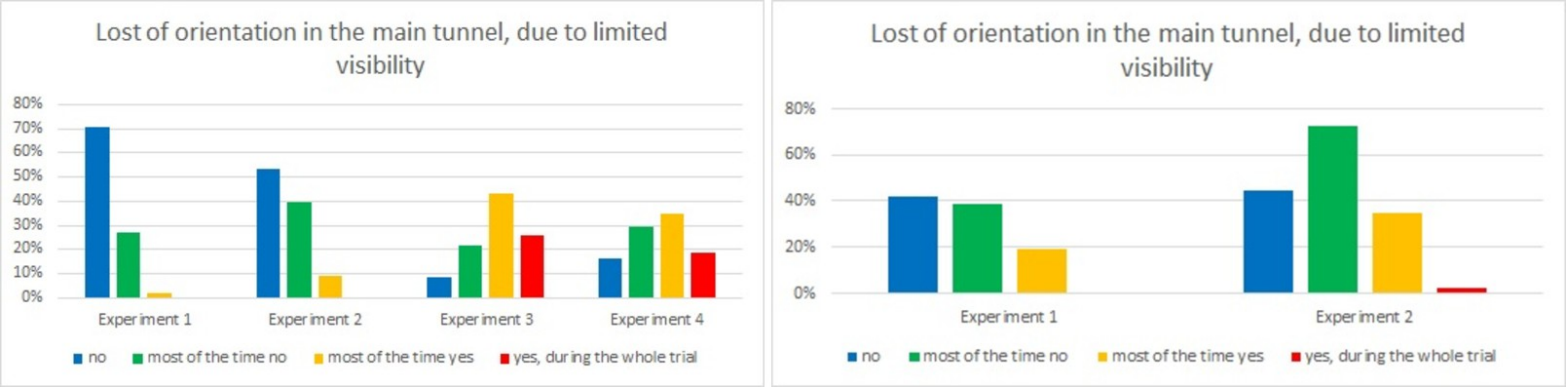
Problems with bearing, due to limited visibility—question 5. (Results from Laliki experiment are on the left side, from Gdańsk on right).

Questions 4 and 5 are related to visibility loss. In question 4, we asked about the decrease in visibility observed (Fig 13), while question 5 asked about lost bearing due to smoke (Fig 14). In question 4, in Laliki the tunnel, one can observe clear differences between Experiments 1 and 2 versus Experiments 3 and 4. During Experiment 1, most participants did not observe visibility loss during most of the evacuation (66%); during Experiment 2, the result was similar (54%). However, in this case, a greater proportion of the students answered most of the time yes (28%). This answer was the most popular in Experiment 3 and Experiment 4 (55% and 49% respectively). Additionally, the number of participants who answered yes significantly increased, during the whole experiment of Experiments 3 and 4 (28% and 15% respectively). Surprisingly, this percentage is higher for Experiment 3 than 4. In Gdańsk, the majority of evacuees answered that most of the time they observed a decrease in visibility on the evacuation path either in the first or second trial (66% and 55% respectively).

In question 5, in Laliki, one can observe a similar difference between Experiments 1, 2 and 3, 4 as in the previous question. During Experiment 1, the pedestrians did not lose their bearing in the tunnel due to smoke at all (70%) or most of the time (27%): for Experiment 2 it is 54% and 40% respectively. On the other hand, during Experiment 3, 43% of evacuees claimed that they lost their bearing most of the time, or during the whole experiment (26%). The results in Experiment 4 are similar: evacuees lost their bearing only slightly less frequently. In Gdańsk, 42% individuals claimed that they did not lose their bearing in Experiment 1 at all (42%) or most of the time (39%); for the second trial, it is 54% and 72% respectively. Loss of bearing most of the time was more frequent in Experiment 2 than in Experiment 1 (35% and 19% respectively).

**Fig 15:**
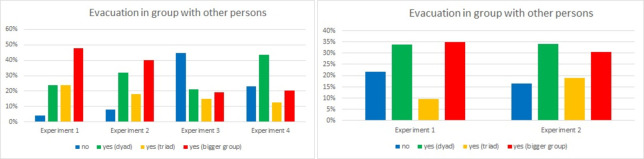
Grouping behavior during consecutive experiments—question 6. (Results from Laliki experiment are on the left; results from Gdańsk on the right).

In question 6, participants were asked if they evacuated in groups (Fig 15). During the Experiment 1 in the Laliki tunnel, almost half of the students evacuated in bigger groups (48%), while the same number of students walked in twos and threes (24% each), and only 4% went alone. In the second experiment, one can observe similar results, with a certain shift from threes and bigger groups to twos. Grouping behavior changed completely during Experiment 3 (competitive, low visibility). Here, almost half of participants evacuated alone (45%). Finally, in Experiment 4 (new place, low visibility), most evacuees evacuate in pairs (44%). In Gdańsk, one can observe a similarity between Experiments 1 and 2. During the Experiment 1, 35% evacuated in a bigger group while the same number of individuals evacuated in twos, 10% in threes and 22% alone. In the Experiment 2, the results were 31%, 34%, 19%, 16% respectively. These results clearly show the influence of external conditions on grouping behavior.

### 3.3 Evacuees’ well being

**Fig 16:**
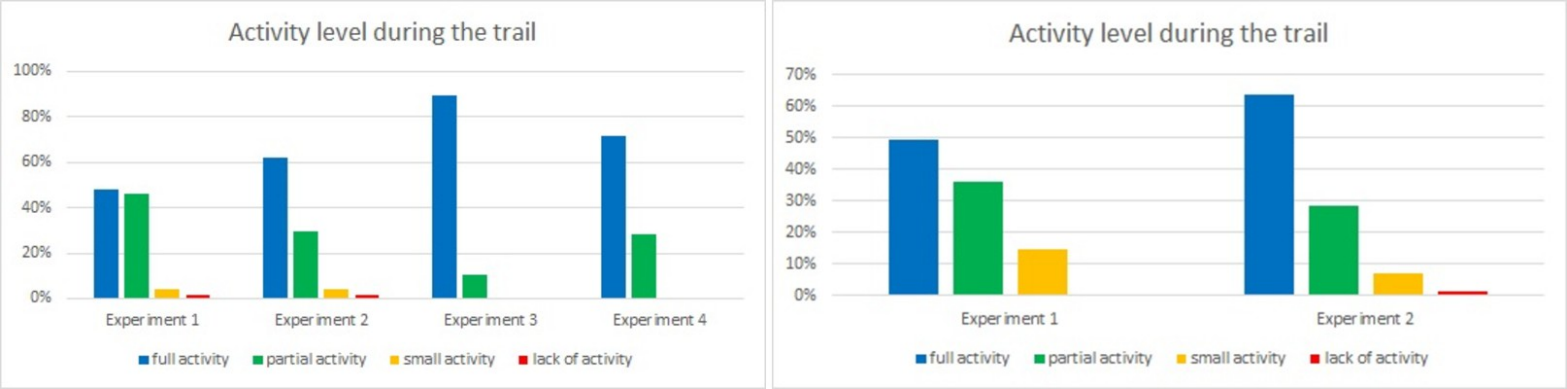
Evacuees activity level during consecutive experiments—question 7. (Results from the Laliki experiment are on the left; results from Gdańsk are on the right).

**Fig 17:**
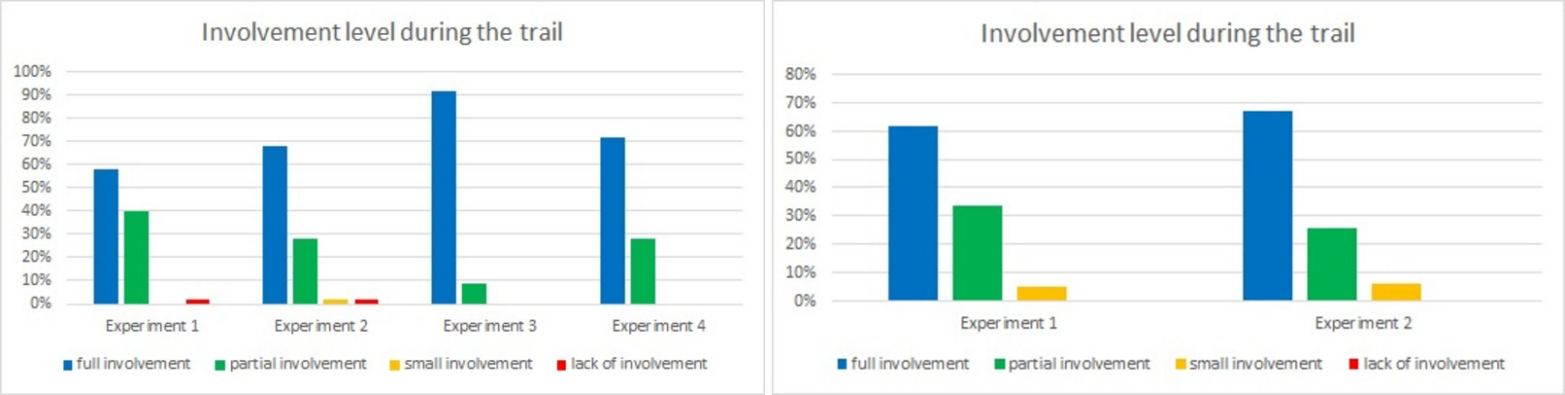
Evacuees’ involvement level during consecutive experiments—question 8. (Results from the Laliki experiment are on the left; results from Gdańsk are on the right).

One set of questions was dedicated to verifying participants’ well-being during experiments. Results for questions 7 and 8 were similar in both tunnel experiments (Figs 16 and 17): we asked participants to rate their activity and involvement level respectively. The highest rating for both of them was observed in the Laliki tunnel in the third experiment: almost 90% of respondents’ indicated full activity and full involvement. The lowest, but still high, results were observed in Experiment 1, both in Laliki and in Gdańsk. It is important to note that only a few respondents indicated little activity/involvement, and hardly any indicated a lack of activity/involvement.

**Fig 18:**
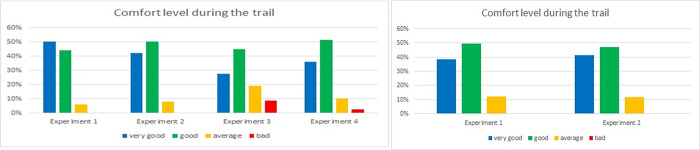
Participants’ comfort level—question 9. (Results from the Laliki experiment are on the left; results from Gdańsk are on the right).

The comfort level of participants during consecutive experiments was investigated in question 9. In the Laliki tunnel, despite the fact that they found themselves in a new, unexpected situation, they felt the most comfortable during Experiment 1 (50% of answers were very good, and 44% were good). The lowest level of comfort was observed in Experiment 3 (heavy smoke, competitive evacuation): 28% very good, 45% good, 19% average, 8% bad (for detailed results see Fig 18). In Gdańsk, the results are almost the same in both trials, with a slightly higher comfort level in Experiment 2 (39% very good, 49% good, 12% average, 0% poor) than in Experiment 1 (41% very good, 47% good, 12% average, 0% poor). Answers in this question clearly corresponds with results from question 3 (fear or uncertainty—Fig 12).

**Fig 19:**
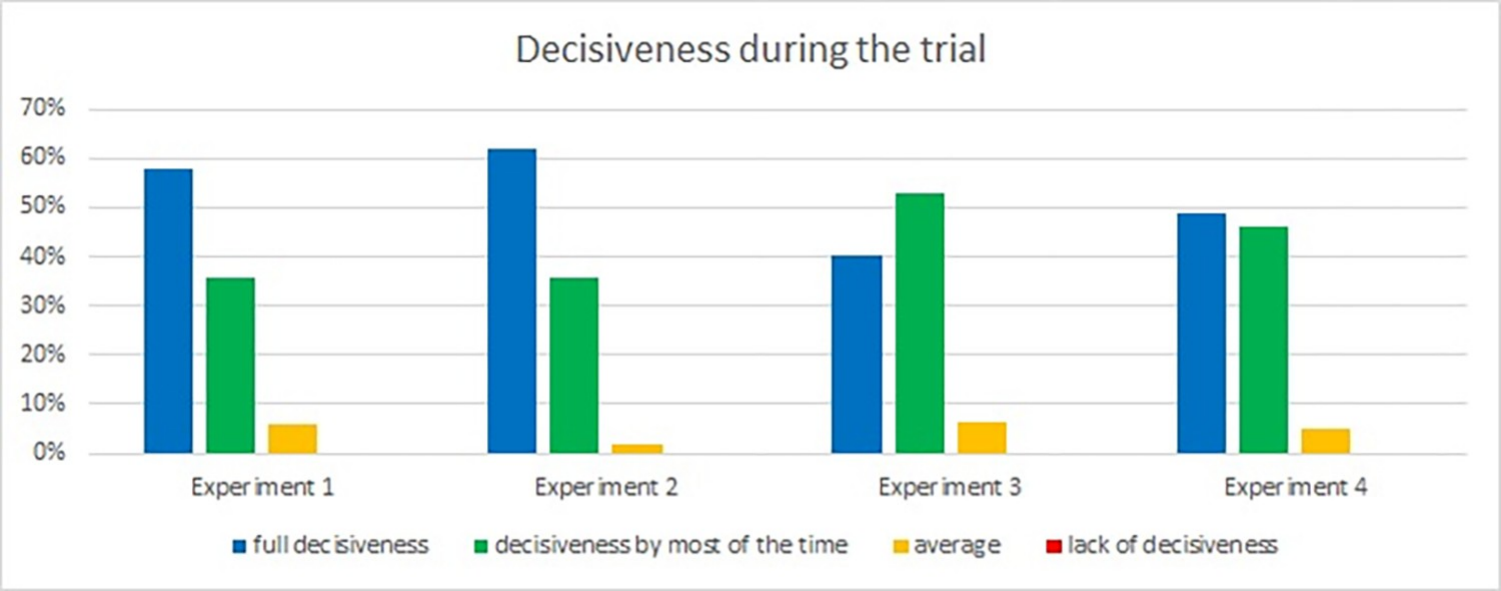
Decisiveness rating—question 10. (Results from the Laliki experiment are on the left; results from Gdańsk on the right).

Finally, we asked the passengers about their decisiveness during experiments (Fig 19). The results from both tunnels were quite similar for all experiments. However, in Laliki, there were some differences between Experiments 1,2 and 3,4. During the last two experiments, participants indicated a lower level of decisiveness in two last experiments. In Gdańsk, the level of decisiveness was slightly higher in the second trial, which may have been related to a higher comfort level.

### 3.4 Participants’ observations

In each survey, participants could describe their observations. From both tunnel experiments, we have gathered in a total of 201 comments. In order to systematize this data, the author have has assigned some categories to each comment/part of the comment.

**Fig 20:**
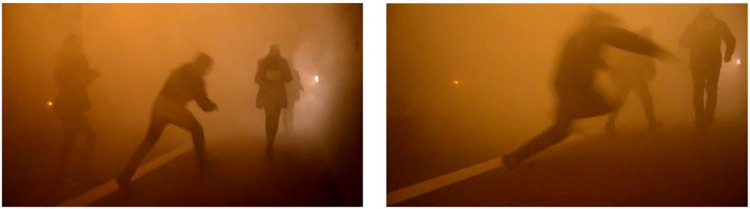
Examples of participants tripping on the curb and falling during Experiment 4 in Laliki.

In the Laliki tunnel, among all comments, the most popular are remarks about unmarked curbstones (20 appearances). However, such remarks were only made during heavy smoke in Experiment 3 and 4; at earlier stages, this issue was not mentioned. Students observed that this lack of curbstone marking *slowed them down and increased their feeling of uncertainty*. Due to very poor visibility (Cs~1.0–1.1m^-1^), the curb became a serious obstacle during the evacuation. Several participants tripped on the curb (Fig 20) and some ran into one another.

18 comments on low audibility of pre-recorded alarm information were also received. Respondents reported echo and reverberation appearance as the source of the problem. In the main tunnel, one has to listen very carefully to understand instructions. Moreover, participants claimed that one can only hear an unclear noise inside the bus; similar remarks have been received in the other experiment [31]. This comment appears mostly during Experiment 1 and Experiment 2. It may be explained by the fact that after two experiments, participants finally learn what is recorded and can understand the message.

Participants’ observations on escape route marking were both positive (9 comments) and negative (18 comments). It is worth noting that positive opinions were received mostly after Experiment 1 (4 comments) and Experiment 2 (3), while negative comments were received mostly after Experiment 3 (15). This may indicate that escape route marking is suitable for clear air or even moderate smoke, but needs to be improved in case of heavy smoke. Some participants also point out that signs showing distances to the nearest evacuation passage are somehow misleading because they show arrows pointing in both directions.

The comments received also show the importance of experience in such a situation. After the first experiments, few observations were made with regard to *indecisiveness* and *tardiness in starting the evacuation*. On the other hand, after the second experiment, we received 7 comments that show the learning effect on the decision to start an evacuation and the choice of escape path. The influence of the”fire evacuation instructions” was mentioned four times–mostly with regard to bending during evacuation in smoke.

Participants complained about low visibility in the main tunnel (8 times, mostly after Experiment 3). They wrote comments such as: *frightening feeling of lack of visibility*, *it was hard not to fall*, *I had to follow my intuition*. Evacuees also complained about the fact that during the test only one door of the bus was opened. Interestingly, there are the same number of comments (8) expressing a feeling of both fear and calm during the evacuation.

Finally, after the first and second experiments, 13 students described them as *an interesting and useful experience*.

In the Gdańsk experiment, the most popular remarks (there were 23 of them) concerned personal experience. Participants described the experiments as very useful and realistic: *everything was very realistic*, *it felt as if there was a real fire*.

However, participants also complained about low visibility—4 times after Experiment 1 and 8 times after Experiment 2 (when the level of smokiness was high). They wrote comments such as: *poor visibility disturbed us*, *aroused fear and uncertainty*, *it was hard to know which way to go to the exit*, *after leaving the bus there was no visibility*.

Moreover, participants’ observations (11 comments) on escape route marking were negative. Evacuees pointed out that *the signs were invisible*, there were *no flashing emergency exits when there was smoke*, *no marking on asphalt*, *which could be helpful in heavy smoke when you lose your bearing*. Some participants also complained about the fact that there was no information as to which direction to choose after leaving the vehicle. They suggested that there should have been some information in the tunnel as to which end of the tunnel was closer. Evacuees also indicated that there should have been a sign inside the emergency cross passages exit, showing them the distances to the portals, so that evacuees knew which was the shortest way.

Five comments indicated the importance of the learning effect on the decision to start evacuating and choice of escape path in the second trial. The comments show that this experience may increase awareness of how to act in another situation requiring evacuation.

## 4 Discussion

In this research, the individual’s perception, behavior and decision making during the evacuation process have been analyzed on the basis of surveys conducted during two real scale evacuations under smoky conditions. After the evacuation experiment, the surveys were conducted directly after each trial, in response to questions related to: decision making during the evacuation, evacuee wellness, loss of bearing under smoky conditions, evacuation in groups, the influence of smoke on individuals’ behaviour etc. The results showed that:

- Participants started evacuation mostly due to smoke in the tunnel (Laliki: 94%, Gdańsk: 71%) and the fire drill (including the fire siren and voice message), (Laliki: 72%, Gdańsk: 71%).
- The main reason for choosing a particular evacuation path selection is the evacuation signs (Laliki: 90%, Gdańsk: 68%); in the second place were voice alarm messages (Laliki: 34%, Gdańsk: 40%) and in third place was mimicking other people (Laliki: 28%, Gdańsk: 27%).
- When smoke levels were high (Cs ~ 0.8–1.1m^-1^), participants felt fear or uncertainty most of the time (Laliki 32%, Gdańsk 12%).
- Evacuees observed a decrease in visibility on the evacuation path when smoke levels were Cs > 0.7 m^-1^, (Laliki 83%, Gdańsk 81%).
- Pedestrians lost their bearings in the tunnel when smoke levels were high Cs > 0.7 m^-1^, (Laliki 69%, Gdańsk 37%).
- During the first trial of two experiments, when there was an element of surprise and the tunnel infrastructure was unknown and there was no instruction as to what to do, the majority of participants evacuated in a bigger group (Laliki: 48%, Gdańsk 35%).
- Under the smokiest conditions (Cs ~ 1.0–1.1m^-1^) participants evacuated in twos (Laliki 44%).

Evacuees’ activity (e.g. Laliki 90% for experiment 3, Gdańsk 64% for experiment 2) and involvement level during all experiments (e.g. Laliki 91% for experiment 3, Gdańsk 67% for experiment 2) were mostly full or sometimes partial, which corresponded with results of the comfort level (good or very good e.g.–Laliki 83% for experiment 3, Gdańsk 88% for experiment 2) and decisiveness (full or by most of the time—e.g. Laliki 94% for experiment 3, Gdańsk 86% for experiment 2). It should be noted that there was a difference between the impact of evacuation signs and voice alarm messages, which was significantly less important during path selection. Interestingly, for these two questions, the survey results were contrary to the observations described in [22]. Participants claimed that they had made the decision to start evacuating because they saw smoke around the bus (Fig 6) and on the evacuation path (mainly on the evacuation signs) (Fig 7), while observing their behavior during the experiments shows that, for the majority of them, it was more like following the crowd than an individual decision. Similar reasons are given in [31]. This inconsistency may be due to the fact that participants underestimated the actual influence of the group on their behavior or just have related to further checking of path in evacuation tunnel. It is worth mentioning that both voice alarm messages and evacuation signs were beneficial for the improvement of the evacuation [25]. Interesting findings are presented in [32], where it was shown that even if the sign is detected, there are always some evacuees who decide to follow the others rather than the sign. However, it should be emphasized that the guiding systems affect the individuals and the leader [33], which in turn affects the evacuation behavior of the crowd.

On the basis of the survey results, it can be concluded that lack of visibility is the most likely reason for fear and discomfort than an unexpected situation and sudden urge to evacuation (Fig 12). The outcomes observed in the abovementioned experiment 4 in Laliki show that the loss of visibility led to a loss of orientation, which made the participants choose the evacuation route towards the source of the fire. These results correspond with [32] where it was shown that the smoke level is a critical factor determining whether people take a risky route for evacuations. In such circumstances, the evacuation signs may be of little help. When there is dense smoke resulting from a fire, the voice announcements could guide the evacuees out of the tunnel even in the conditions of limited visibility. The research on the efficiency of various types of voice signals is presented in [7].

When describing their own perception of the experiment in Laliki, participants mentioned unmarked curbstones, which they said could be a dangerous obstruction during evacuation under very smoky conditions. Several participants tripped on the curb (Fig 20) and some ran into one another (Experiment 4). Low audibility of pre-recorded alarm information like echo or reverberation appearance was the next problem indicated by evacuees in Laliki tunnel. This is an important element of tunnel infrastructure used for starting an evacuation, especially when people need clear information in unexpected situations like fire in a tunnel. Otherwise, they might be indecisive and slow to start evacuating–and this was the next issue mentioned by participants after Experiment 1 in Laliki.

The next observation was a negative opinion on escape route marking under very smoky conditions, claiming the signs showing the distances to the nearest evacuation passage were misleading (comments for Laliki and Gdańsk). The escape route was the most significant information during path selection, and some passengers said it should be improved for use under very smoky conditions (by lighting up one arrow pointing in only one direction, away from the fire). Similar findings are indicated in [15]—there are some side effects of alarms, and ambiguity of signs, which might potentially result in serious consequences.

During the post-evacuation interview in Gdansk, where the tunnel is bidirectional and has two naves, the participants pointed to a number of signs that are lacking in the infrastructure. Firstly, there was no information about the direction which should be taken after disembarking the vehicle. Secondly, once evacuees enter the emergency cross passage exit, which direction should be taken to exit the tunnel. What is more, there should be road signs indicating the distance to the nearest portal and the emergency exists. Participants also expressed their concern about the safety of crossing over to the second tube and whether they might be hit by the vehicles there.

Many comments expressed a positive opinion on the experiment itself, which participants found very useful, realistic and interesting. The role of the learning effect after each subsequent experiment was also emphasized.

In the light of above, the research demonstrates the basis on which the pedestrians undertake the evacuation, the course of the evacuation, what influences the choice of the evacuation route and the reception of the technical installations—which are vital to the process of evacuation. Moreover, the reaearch gives a comprehensive understanding of the relation between the tunnel infrastructure and pedestrian evacuation needs. Based on these findings, the tunnel authorities can have an insight and enhanced understanding of the particular factors that impact the safety of the evacuation, such as curb markings, improving the quality of voice announcements, enabling the evacuation of people on wheelchairs, road signs, and adding the signs informing about the emergency exits and the distance towards portals in the second tube. The approach proposed in [32] regarding the wider use of colors in the surrounding infrastructure that influence their perception, is of particular interest.

On the basis of the outcomes collected from both experiments and the literature review, it seems reasonable to use red illumination of the door in the tunnel for the evacuation passage located in the immediate vicinity of the fire (which is supposed to suggest a threat), and green illumination of those exits that are safe, giving a clear message on how to safely choose the direction of the escape route. According to [25], the induction effect of exit outline lamps and unilateral lamp strips is the best, which has the most obvious effect on increasing the identification of exit.

The results of this kind of research, based on real scale evacuation experiments in road tunnels, are necessary for increasing safety in road tunnels and especially for a better understanding of evacuees’ behavior and improving tunnel infrastructure as well as the calibration and validation of individual parameters in computer models of evacuation or risk assessment. However, the issue require continuation and conducting new research, taking into account participants of various range of ages, which may influenced into different factors like: the decision-making process and the speed of movement. There are also some factors which the experiment does not include; due to the cold, non-toxic smoke it was impossible to measure the participants’ reaction towards the heat and radiation which are characteristic of real fire. Owing to the safety reasons, such analysis could not have happened. Therefore, the behavior of the participants did not reflect the exact reaction they might have during the real fire. However, the data provided by both experiments are a valuable reference for the understanding of psychology and behavior of people during the evacuation emergency in partially closed spaces.

## Supporting information

S1 Appendix (DOCX)
