# Supplementary material for: Subjective individuals’ perception during evacuation in road tunnels: Post-experiment survey results
Source: PLoS One. 2023 Mar 30;18(3):e0283461. doi: 10.1371/journal.pone.0283461 (PMC10062673; doi:10.1371/journal.pone.0283461)
Supplement: S1 Appendix — (DOCX) [file pone.0283461.s001.docx]

**Appendix**

**SURVEY**

## **General data:**

| ID number: |  |
| --- | --- |
| Age: |  |
| Gender: |  |
| Year and discipline of studies: |  |
| Height: |  |
| Weight *(approximate):* |  |
| Shoulder width: |  |

1. **Have you ever participated in a trial or real tunnel evacuation?**

| - 1. YES | - 1. NO |
| --- | --- |

1. **Have you ever participated in an evacuation?**

| - 1. YES | - 1. NO |
| --- | --- |

1. **Have you ever tried to move/evacuate in smoky conditions?**

| - 1. YES | - 1. NO |
| --- | --- |

1. **Do you know the exact code of conduct in the case of fire in a road tunnel?**

| - 1. YES | - 1. NO | - 1. partially |
| --- | --- | --- |

| ID numer: |  |
| --- | --- |

1. **Choose one or two basic reasons for deciding to start evacuating:**

| *A - bus stopping* | *B – fire drill* | *C – smoke in the tunnel* | *D – other people’s behavior* |
| --- | --- | --- | --- |
| *E – other :………………………………………………………………………………………* | | | |
|  | | | |

1. **Choose one or two basic basic reasons for choosing a particular path during evacuation:**

| *A – mimicking other people* | *B- evacuation signs* | *C – voice alarm messages* |
| --- | --- | --- |
| *D – intuition,* | *D - knowledge of evacuation procedures* | *F – previous experiences* |
| *H - other …………………………………………………………………* | | |

1. **Did you feel fear or uncertainty during the evacuation?**

| *A – no* | *B – most of the time no* | *C – most of the time yes* | *D – yes, during whole trial* |
| --- | --- | --- | --- |

1. **Did you observe a decrease in visibility on your evacuation path?**

| *A – no* | *B – most of the time no* | *C – most of the time yes* | *D – yes, during whole trial* |
| --- | --- | --- | --- |

1. (Only if you have choosen B, C or D in question 4)
   **Did you lose your bearing in the main tunnel due to limited visibility?**

| *A – no* | *B – most of the time no* | *C – most of the time yes* | *D – yes, during whole trial* |
| --- | --- | --- | --- |

1. **Did you evacuate in a group with other people ?**

| *A – no* | *B –* yes (twos), | *C –* yes (threes), | *D –* yes (bigger group) |
| --- | --- | --- | --- |

1. **Rate your activity level during this trial.**

| *A – full activity* | *B – partial activity* | *C – low actvity* | *D – lack of actvity* |
| --- | --- | --- | --- |

1. **Rate your involvement level during this trial.**

| *A – full involvement* | *B – partial involvement* | *C – little involvement,* | *D – lack of involvement* |
| --- | --- | --- | --- |

1. **Rate your comfort level during this trial.**

| *A – very good* | *B - good* | *C - average* | *D – bad* |
| --- | --- | --- | --- |

1. **Rate your decisiveness during this trial.**

| *A – full decisiveness,* | *B - decisiveness by most of the time,* | *C - average,* | *D – lack of decisiveness* |
| --- | --- | --- | --- |

1. **Rate the audiability of alarm informations:**

| A – excellent audibility | B – very good audibility | C- good audibility |
| --- | --- | --- |
| D – passable audibility | E – very weak audibility | F – no audibility |

1. **Rate escape route marking:**

| A – excellent marking | B – very good marking | C- good marking |
| --- | --- | --- |
| D – passable marking | E – very week marking | F – unnoticeable marking |

1. **Describe your observations and impressions from this trial.**
